# A Two-Phase Model for Smoothly Joining Disparate Growth Phases in the Macropodid *Thylogale billardierii*

**DOI:** 10.1371/journal.pone.0024934

**Published:** 2011-10-12

**Authors:** Clive R. McMahon, Marie-Jeanne Buscot, Natasha L. Wiggins, Neil Collier, John H. Maindonald, Hamish I. McCallum, David M. J. S. Bowman

**Affiliations:** 1 Research Institute of Environment and Livelihoods, Charles Darwin University, Darwin, Australia; 2 School of Plant Science, University of Tasmania, Hobart, Australia; 3 Centre for Mathematics and its Applications, Australian National University, Canberra, Australia; 4 Griffith School of Environment, Environmental Futures Centre, Nathan campus, Nathan, Griffith University, Queensland, Australia; University of California, Berkeley, United States of America

## Abstract

Generally, sigmoid curves are used to describe the growth of animals over their lifetime. However, because growth rates often differ over an animal's lifetime a single curve may not accurately capture the growth. Broken-stick models constrained to pass through a common point have been proposed to describe the different growth phases, but these are often unsatisfactory because essentially there are still two functions that describe the lifetime growth. To provide a single, converged model to age animals with disparate growth phases we developed a smoothly joining two-phase nonlinear function (SJ2P), tailored to provide a more accurate description of lifetime growth of the macropod, the Tasmanian pademelon *Thylogale billardierii*. The model consists of the Verhulst logistic function, which describes pouch-phase growth – joining smoothly to the Brody function, which describes post-pouch growth. Results from the model demonstrate that male pademelons grew faster and bigger than females. Our approach provides a practical means of ageing wild pademelons for life history studies but given the high variability of the data used to parametrise the second growth phase of the model, the accuracy of ageing of post-weaned animals is low: accuracy might be improved with collection of longitudinal growth data. This study provides a unique, first robust method that can be used to characterise growth over the lifespan of pademelons. The development of this method is relevant to collecting age-specific vital rates from commonly used wildlife management practices to provide crucial insights into the demographic behaviour of animal populations.

## Introduction

Describing the growth of animals over their entire life is a fundamental component of wildlife and conservation management [1], [2] because growth is a reflection of the intrinsic (competition) and extrinsic (environmental) variability to which animals are exposed to and it determines many critical life-history parameters of individuals (e.g. the onset of breeding (primiparity) [3] and their survival probabilities [4]). Moreover, the ability to quantify what appears to be a simple relationship between two variables (e.g. time or age and some other variable such as length, height or mass) provides invaluable information that can be used to model and predict the behaviour of entire populations [5] under natural conditions and their responses when they are subject to management interventions (e.g. harvest quotas).

Macropods and generally most eutherians, typically display two distinct growth phases during their lives [6], [7] broadly characterised as the ‘pouch’ and ‘post-pouch’ phases. Pouch growth rates are typically greater than post-pouch rates and consequently conventional sigmoid curves can be used to describe life-time growth, with varying success [6]. In macropods specifically, growth rates decrease relatively abruptly once the young vacate the pouch [7]. In addition to this abrupt rate change, the variation in individual body mass and morphometric measurements increases markedly among animals in the post-pouch phase because of shifts in diet of the weaning and weaned animals, the increased energetic expenditure associated with being out of the pouch environment, and the additional stressors associated with foraging success, competition, onset of reproduction and predation pressure [8]. These varying rates of growth are problematic when trying to fit ‘standard’ growth curves (e.g. Logistic, Brody and Richard's growth functions) that describe the relationship between age and morphometric traits such as tail length, leg length and head length, with the latter two measurements being the most reliable [9], [10]. Single growth curves tend to poorly describe these growth patterns: the models might describe one phase of growth well but provide less predictive power when the growth phase shifts according to the change in life-history stage. This is to be expected as these types of models do not account for the change in growth phase. In order to accurately model the phase change in growth, two of these ‘standard’ curves are required, one describing pre-weaning growth and the other describing post-weaning growth [9], [11]. These two growth curves can, at least for the data and curves that we have used, be made to join smoothly.

Macropods are well adapted to Australia's variable and extreme climates and they are abundant and ubiquitous species across the many and varied landscapes in Australia. The conspicuous and charismatic species, such as kangaroos and wallabies, like all macropods, can respond rapidly to the variable environmental conditions in Australia and have been little disrupted by European agricultural practices. In fact, some species of macropods have arguably responded positively to the presence of modern agriculture and are now considered major pests due to their abundance and interactions with humans and agriculture [12]. Each year, tens of thousands, possibly millions, of macropods are culled and harvested to limit direct damage to agriculture and the indirect damage to grazing industries caused by competition between native macropods and introduced grazing herds. One such macropod is the endemic Tasmanian pademelon (*Thylogale billardierii* Desmarest 1822). In Tasmania, an estimated half a million Tasmanian pademelons are culled annually to reduce their impact on agricultural, grazing and forestry practices [13]. The intended outcome of this *ad hoc* intervention is the long-term reduction in population densities to reduce negative grazing impacts on pastures and forestry coups. However, to effectively achieve long-term reductions of population densities, the management of this species needs to be based on knowledge of their basic life-history patterns (e.g. age structure, diet and movement patterns) to determine optimal culling strategies – those that reduce densities and restrict reinvasions [5], [14]. Of particular importance is the ability to accurately age individual animals across the age spectrum of the population [15]. Categorising individuals into age classes allows one to calculate demographic information such as vital rates (e.g. survival, mortality, fecundity) which can then be used to construct population models [5] and develop the culling strategies required for efficient management [16]–[19].

**Figure 1:**
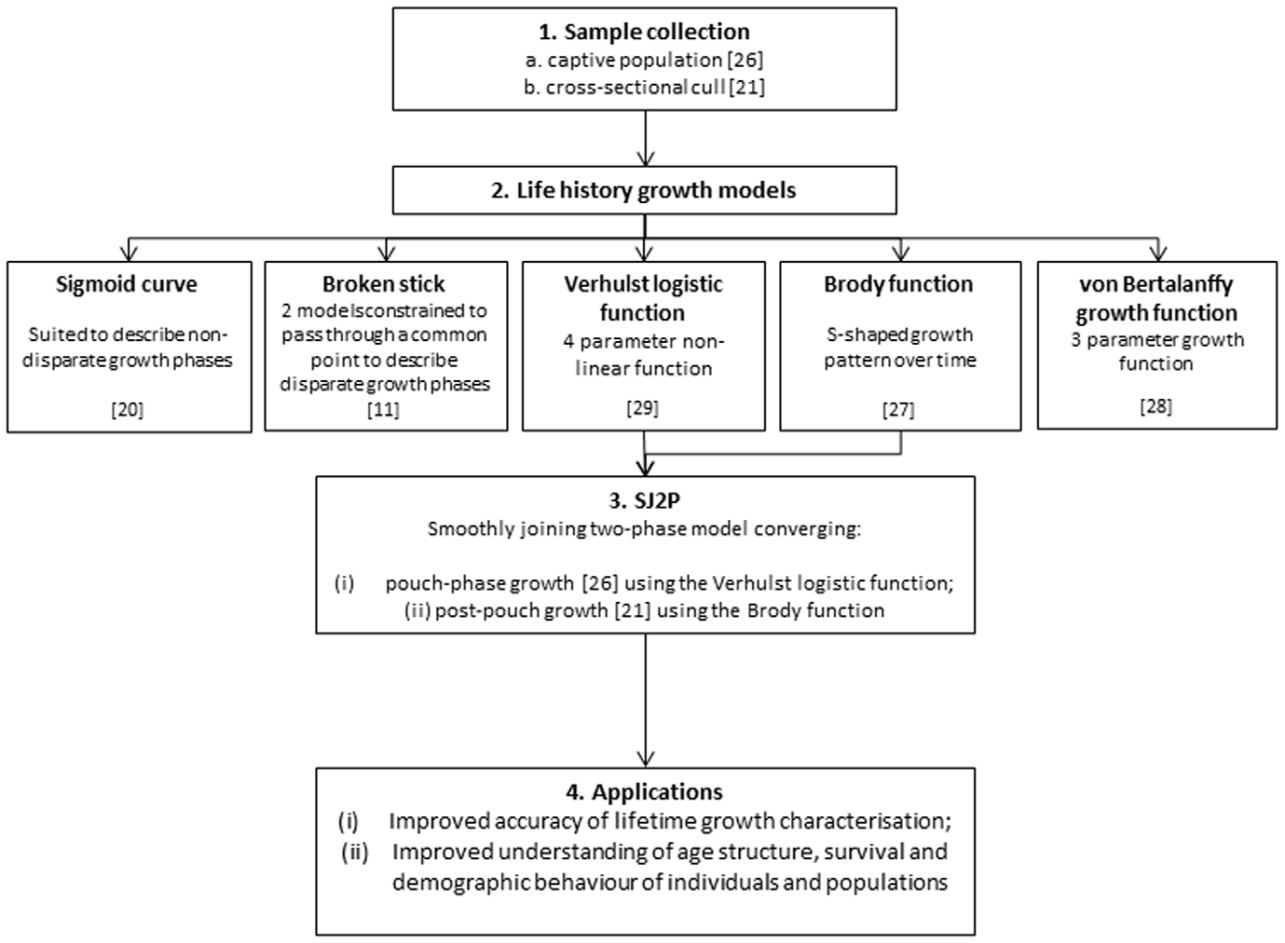
Overview of research aims, where (1) Sample collection involved head and foot (pes) measurements from (a.) a captive population of pouch-young pademelons [**25**] and (b.) a cross-sectional cull of a single cohort of pademelons [**20**] followed by (2) model selection for describing the life history growth of pademelons; followed by the development of (3) a smoothly joining two-phase (SJ2P) model; for (4) the improved accuracy of characterising and describing the lifetime growth of animals with disparate growth phases in order to enable an improved understanding of the age structure, survival and demographic behaviour of individuals and populations.

Here, we present a novel approach for quantifying the lifetime growth of the Tasmanian pademelon, as a test case, that is an advance on the application of broken-stick modelling approaches used to describe relationships when there are relatively abrupt growth transitions over time. Our aim was to build upon the applications of a quasi-single model used in broken-stick modelling to develop a real-single, converged element model to provide a tool to age animals with disparate growth phases, in order to provide a more accurate description of their lifetime growth (Fig. 1).

## Materials and Methods

### Ethics Statement

Approval for research was granted by the University of Tasmania Animal Ethics Committee (Permit Number A9895) and the Parks and Wildlife Service (Permit Numbers 2803362 and 2801251).

### Study site

Research was conducted in the north Scottsdale region located in north east Tasmania, Australia (41°06′S; 147°35′E). The study site was concentrated around two agricultural properties, spanning a greater area of 285 ha. Two habitat types dominated this site: (1) dry eucalypt forest and woodland; and (2) agricultural land [21].

### Sampling

A cross-sectional cull of macropods occurred over an 18 day period, from January 27 to February 13 2009. The cull was performed over a total of nine nights during this timeframe; four nights at Property 1 and five nights at Property 2 [21]. In total, 230 individuals (Tasmanian pademelons) were obtained for study from this cross-sectional cull.

### Morphometric Data Collection

Morphological measurements (body weight, head length, jaw length, ear length, body length, tail length, tail circumference, pes length) and reproductive measurements (sex, testes length and width, or foetus length and sex) were taken at the time of the cross-sectional cull. Macropod heads were collected, sealed in plastic zip-lock bags, frozen and transported to the University of Tasmania. At a later date, these samples were placed in concentrated bleach (White King® concentrated bleach with 63.0 g/L sodium hypochlorite [available chlorine 6.0% m/v]; Sara Lee, Pymble, Australia) for 24 hours then aged using molar eruption sequences [22]–[24]. Head measurements were taken as the distance from the foramen magnum to the end of the rhinarium, pes as the length of the plantar surface of the hind foot minus the nail, and tail as the length of the underside of the tail minus any hair extending from the tip as described by [25]. Additional head, pes and tail length data previously collected on 68 captive Tasmanian pademelons, aged between 20 days to 12 months, were included in our analysis [26]. These additional data allowed us to describe the animals' growth over their entire life span. The same measurements of head, pes and tail length were recorded; however, the genders of the animals were not recorded. While sexual differentiation was noticed to occur at the early developmental stage, allowing gender determination at about 14 days, individuals younger than 7 months displayed little growth rate variability as long as they remained in the stable pouch environment. This confirms observations that there are no observable differences in growth rate between the sexes [22], until the animals are more than a year old – during the late weaning period – before which males and females are classified together in a so-called indifferent stage [25], [26].

### Model fitting – previously used models

Here we follow the convention of fitting two non-linear curves to the data: one describing growth up to 207 days, when the young pademelons typically vacate the pouch [26], and growth after 207 days. Vacation of the pouch marks the beginning of the weaning period during which the joey stays at-foot of the mother and returns to the pouch to suckle: complete weaning occurs between 8 and 12 months.

In other macropodidae (including the agile wallaby, the Yellow footed Rock-wallaby, the red, the grey and the eastern kangaroo), the growth of any body measurement (m) is characterised by a 4-parameter non-linear model consisting of two hyperbolic curves constrained to pass through a common point (b4) at the time when the young vacate the pouch. These hyperbolic curves are defined as:
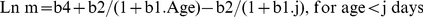
(1)

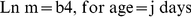
(2)

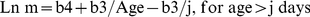
(3)where m is the body measurement in millimetres or weight in grams, Age is the age in days, b1, b2, b3 and b4 are the growth parameters and j is the age at which the young vacates the pouch. The fitted value for body measurement at age j is given by b4 and the absolute value of b3 is related to the growth rate (ln) after vacation of the pouch [11]. The relationship between head length and age after vacation of the pouch has not been previously investigated.

### Model fitting – models considered for the current data

We attempted to fit the two hyperbolic curves commonly used to describe the two phases of growth in other macropodidae [11] to the pes length and head length at age data collected. For comparison with the smoothly joining two-phase (SJ2P) model that will be described shortly, we also preliminarily fitted the single Brody function [27] growth curve to the entire range of pes length measurements (i.e pre- and post weaning individuals considered together).

The SJ2P model was constructed using the following individual growth models frequently used to describe body growth in animal ecology: the von Bertalanffy [28], the Brody [27] and the Verhulst logistic [29] growth curves. These models were fitted separately to the morphometric data of either the pouch or post-pouch growth phase data (R [30]–[31]). The models obtaining the best fit models were joined smoothly to describe growth over the life time of the animal. The entire model fitting was performed using the function nls in R that is designed for fitting nonlinear models [30] (Appendix A). The von Bertalanffy growth (VBGF) is a 3 parameter function defined as:
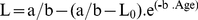
(4)where L_0_ is the mean value of the body measurement at birth (Age = 0), a/b is the parameter that predicts the asymptotic measurement at mature age (lim*_Age_*_→∞_), and b is a constant curve parameter representing the ratio of maximum growth rate to mature size with units of reciprocal time (e.g. year-1), often referred to as maturing rate index. The von Bertalanffy model leads to a linear decrease in growth rate as a function of size and it has no inflexion point. Growth is fastest at the outset and gradually diminishes until it reaches zero. Growth is determinate and size cannot exceed the horizontal asymptote of the curve at *L*(*t*) = *a/b*.

The Brody model [27] describes growth as a conjunction of the monomolecular function with the exponential and thus accounts for an S-shaped growth pattern over time. The Brody model is defined as:
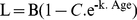
(5)where B is the mature (or asymptotic) body measurement, C is an adjustment parameter when *L0*≠*0* or *t*≠*0*, and k is the maturing rate index representing the ratio of maximum growth rate to maximum size. This model describes growth as “self accelerating” (exponential) before and “self inhibiting” (monomolecular) after a certain age. The Brody growth function thus describes a sigmoidal behaviour but with a discontinuity at the point t = t′ reflecting a shift in the growth pattern. The relative growth rate in the Brody function declines non-linearly as the body measurement L increases [32].

The Verhulst logistic growth [29] is a 4-parameter non-linear function defined as:
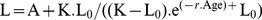
(6)where A is the value of the lower asymptote of the growth curve (A≠0), L_0_ is the value of the body measurement at birth, K is the maximum possible size of the body measurement when lim*_Age_*_→∞_ , and r is the intrinsic growth rate. The relative growth rate, (1/*L*)(d*L*/d*Age*), declines linearly with increasing population size and reaches its zero minimum when *L* = *K*. The body measurement L at the inflection point (where growth rate is maximum), *L*_inf_, is exactly half the maximum possible size, *L*_inf_ = *K*/2, and the maximum growth rate is (d*L*/d*Age*)_max_ = *rK*/4.

For the SJ2P logistic/Brody models we require equations 7 and 6 above, the parameter *A* in Eqn. 7 is determined by equating the values of *L* at the transition point *Age = j*, called critage in the R code. Requiring the curves to have the same slope at that point requires that:
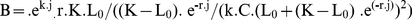
(7)The model fit was evaluated by analysing the adjusted R-squared and the Root Mean Square Error (RMSE), and by plotting the residuals and checking for any pattern. Because of the greatly increased variability during the second growth phase, the statistics should be calculated separately for the two growth phases. The two statistics are based on sums of squares: Sum of Squares Total (SST) and Sum of Squares Error (SSE). The SST measures how far the observed data are from their mean and the SSE measures how far the observed data are from the model's predicted values. The adjusted R- square is a relative measure of fit incorporating the model's degrees of freedom. It is interpreted as the proportion of total variance that is explained by the model. The RMSE incorporates the square root of the variance of the residuals. It indicates the absolute fit of the model to the data: how close the observed data points are to the model's predicted values. The RMSE can be interpreted as the standard deviation of the unexplained variance, and has the property of being in the same units as the response variable. Lower values of the RMSE indicate better fit. The RMSE is a good measure of how accurately the model predicts the response, and is the most important criterion for assessing the predictive power of a model. It has the additional advantage that it is unaffected by the range of values of the response variable.

In the cases where two separate functions were used to describe head and pes growth of the animals, the age j at the join point (end of the pouch life) was first fixed at j = 207 days (range 196–212 days) as previously defined [26]. In order to achieve a better fit, and to refine the estimation of age at pouch vacation, the parameter ‘age j’ was included as a parameter to be estimated in the SJ2P model. The estimated values of the age j were compared to the weaning ages previously observed in Tasmanian pademelons. The join point in the SJ2P model was chosen to minimize the residual sum of squares. As noted earlier, a constraint was imposed that forced the curves to join smoothly (Appendix S1).

## Results

The estimated ages of the culled animals (Tasmanian pademelons) ranged between 17 and 80 months, which is in accordance with previous observations setting the life expectancy of pademelons at about seven years [25]. The culled samples from the Tasmanian population were significantly female biased (with a t-test reporting p≤0.05, 42% of males). Males were on average bigger than females (K-W H (1, N = 120) = 10.88, p = 0.05) and had significantly larger average pes lengths and head lengths than female pademelons (χ^2^ = 12.38, dl = 1, p = 0.02).

### Growth Model fitting during the pouch phase

**Figure 2:**
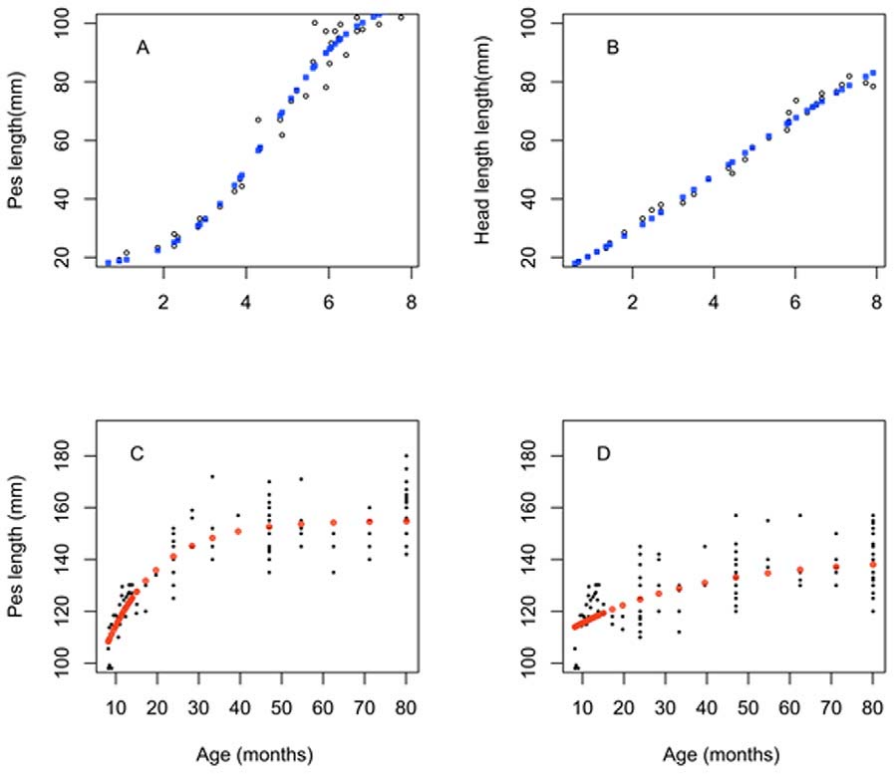
The growth of juvenile Tasmanian pademelons (*Thylogale billardierii*) during the pouch phase of their life-history. Black circles represent (A) pes length and (B) head length of pouch young (mm); blue squares represent the Verhulst logistic model used to describe (A) pes length and (B) head length growth (mm) during pouch life(Length = A+K.L0/((K−L0).e^(−r . Age)^+L0, r^2^ = 0.98). Post-weaned pes length growth is represented for(C) males and (D) females, where black circles represent pes length (mm) of pouch young and aqua squares represent the Brody model used to describe post-weaned pes length growth(Eqn.5). Pes Length = A C; L = *B*. (1−*C*.e^−k . Age^), r^2^ = 0.71, and r^2^ = 0.44 respectively for males and females.

As previously observed [26], there was a strong linear relationship between head length and age during pouch life up to 207 days (H = 0.8643+0.2452A, r^2^ = 0.975) in the captive young animals. However, head length growth up to this same age was also very well represented by the Verhuslt logistic function (r^2^ = 0.98, RMSE = 2.309, Fig. 2).

Pes length growth during the 210 days of pouch life had an exponential tendency that slowed down as it approached 207 days (Fig. 2). We applied the von Bertalanffy growth function (Eqn. 5) to the pes length but the model poorly described the s-shaped growth observed in the young individuals. The pre-weaning growth appeared to be sigmoidal and graphically symmetrical so we fitted a logistic growth function from birth to weaning age, taking into account the non-linear variation of the growth rate over time. The fitted logistic model obtained the best goodness of fit (r^2^ = 0.98, RMSE = 4.91; Table 1) and described well the relationship between pes length and age of the pouch young. A relatively slow growth during the first weeks following birth was observed, followed by an acceleration of the growth rate (maximum at Age = Pouch vacation age / 2), followed by a slight slowdown in growth rate before attaining weaning age. However, and importantly, it was noted that no convergence was reached when trying to fit the previously suggested four parameter relationship (Eqn.1) [9]. Neither pes length nor head length at age data displayed hyperbolic behaviour over the pouch life, indicating that this model was not appropriate for describing the growth of pademelon pouch young.

**Table 1:** Coefficients of the Logistic and von Bertalanffy growth models describing pes length growth of pouch young and post-weaned Tasmanian pademelons (*Thylogale billardierii*).

Model	Life stage	Coefficients					r^2^		RMSE
Logistic		A	K	L0			r		
	P	16.06±3.40	93.92±6.08	1.11±0.684			0.96±0.12	0.98	4.91
von Bertalanffy		a	b	L0					
	P	13.79±2.50	0.022±0.03	2.42±5.289				0.93	7.41
Brody		a	b		k				
Male	E	10.0±6.36	137.0±6.18		1.20e-01±0.08			0.53	14.31
Female	E	10.0±8.32	175.0±8.02		7.49.e-02±0.07			0.62	11.2
Brody		B	C		k				
Male	PP	154.91±1.35	0.567±0.08		0.077±0.013			0.71	9.85
Female	PP	140.55±3.11	0.247±0.02		0.030±0.011			0.44	9.19
SJ2P		C	K	L0	k	j	r		
Male	E	0.57±0.012	95.21±4.42	1.22±0.09	0.078±0.001	7.85±0.02	0.78±0.09	0.95	8.76
Female	E	0.22±0..3	111.42±7.2	4.34±1.05	0.024±0.002	11.56±0.08	0.94±0.07	0.93	8.26

Parameter A is the value of the lower asymptote of the pes length growth curve, L_0_ is the body measurement at birth (age = 0), K is the asymptotic pes length in mm and r is the intrinsic growth rate. Parameter a is the parameter predicting asymptotic pes length and b is the maturation rate. Coefficients of the Brody model describe pes length growth after pouch vacation of pademelons. Parameter B is the mature (or asymptotic) pes length, C is an adjustment parameter when *L0*≠*0* or *t*≠*0*, and k is the maturing rate index representing the ratio of maximum growth rate to mature size. For life stage, P indicates a model that describes the pouch life stage, E represents the model that describes the entire life growth and PP represents the model that describes the post-pouch life stage. Goodness of fit is given by r^2^: r-square value of the regression, and RMSE is the root mean square error. SJ2P is the smooth-joining two phase model.

### Growth Model fitting after pouch vacation

The head and pes measurements of pademelons that had vacated the pouch were obtained from culled animals of potentially different cohorts and exhibited significantly higher variability compared with the pouch young. The variability in body measurements of the weaning pademelons made curve fitting difficult and in both cases we failed to fit the second hyperbola of a broken-stick model described previously [9] to the second growth phase of pademelons (Eqn. 3: Ln m = b4+b3/Age−b3/j, for age>j days). However, the Brody model (Eqn. 5) described the second phase of pes length and head length growth both in males and females (Fig. 2). The goodness of fit obtained for the Brody model was stronger in males than in females, both for pes length (Males, r^2^ = 0.71; Females, r^2^ = 0.44) and head length growth (Males, r^2^ = 0.65; Females, r^2^ = 0.62; Table 1).

### The SJ2P model

**Figure 3:**
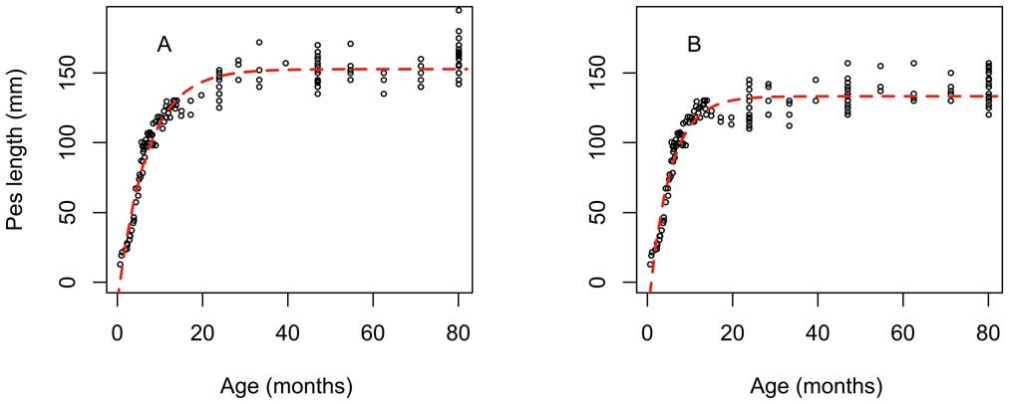
The life-time growth of (A) male and (B) female Tasmanian pademelons (*Thylogale billardierii*) (black circles) and the Brody growth function (black line) fitted on the entire pes length data (pes length = a+b * (1−exp(−k * Age)).

To achieve a description of pes length and head length growth over the whole life span of pademelons, the Logistic growth function and the Brody growth were computed and forced through to join at the fixed age of 8 months for both males and females (join point j = 207 days, Fig. 3). The description of pes and head length were compared, obtained using a single Brody function, with that obtained using the Brody function fitted to the entire pes and head length datasets.

**Figure 4:**
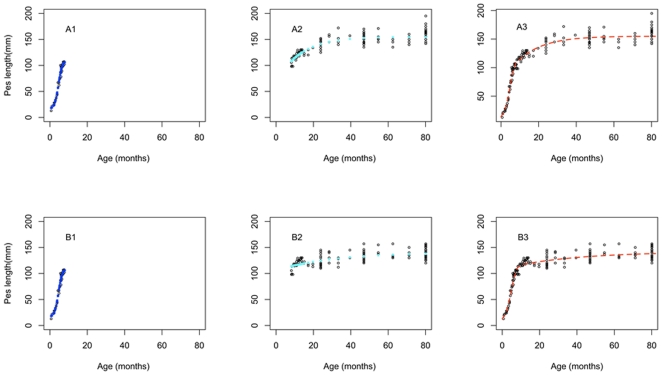
The sequence of steps showing the construction of growth functions for the growth phases of (a) male and (b) female Tasmanian pademelons (*Thylogale billardierii*). Panels a1 and b1 show previously recorded data [25] (black circles) and predicted pes lengths using the Verhulst logistic model (blue filled circles) for pouch young. Panels a2 and b2 show pes lengths of post-weaned animals (black circles) and predicted pes lengths using the Brody growth function (aqua circles) (join point j = 207 days). Panels a3 and b3 show animal age (months) using pes length as the predictor variable, for the observed life of pademelons (black circles) and the converged SJ2P model (black line).

**Figure 5:**
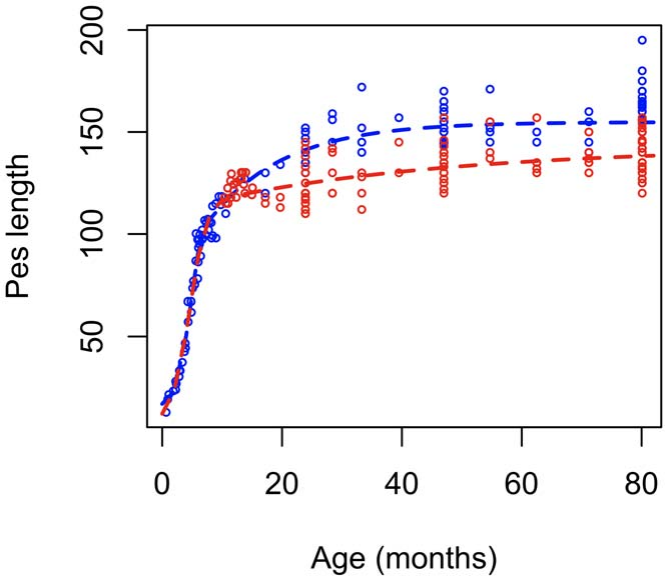
The SJ2P model describes the lifetime growth (pes length) of the Tasmanian pademelon (*Thylogale billardierii*). Red circles denote female pes length and red dashed line shows the SJ2P model describing growth of females from birth to 80 months of age; blue circles denote male pes length and blue line shows the growth of males from birth to 80 months.

The goodness of fit obtained for the single Brody function (Fig. 2) fitted to the entire pes length dataset was much lower (r^2^ = 0.62, RMSE = 11.2 for males, and r^2^ = 0.53, RMSE = 14.31 for females) compared with the SJ2P model consisting of the Logistic and Brody growth functions (r^2^ = 0.93, RMSE = 5.85 for males and r^2^ = 0.78, RMSE = 8.21 for females). Similar observations were true for the description of head length growth, which was more realistic when modelled by the SJ2P model than by the Brody function only (Table 1). The prediction of the age *j* parameter in the six parameter broken stick model (age at which the shift in growth is observed) was close to the age of weaning previously reported [26]. While weaning was observed to occur at 8 months, on average, for the 62 captive animals observed [26], the SJ2P model obtained the best fit for a shift in pes length growth happening at 7.86 months (220.08 days) and 11.56 months (323.68 days) for males and females, respectively (Fig. 4). Similarly, when applied to the entire head length data set, the SJ2P model obtained the best fit for a shift in growth occurring at 8.31 months (232.68 days) for females and 11.64 months (325.92 days) for males (Table 2). This result is similar across both metric estimates used. Results illustrate that male pademelons grow faster than females (Fig. 5).

**Table 2:** Coefficients of the linear and Brody growth models describing head length growth of pouch young and post-weaned Tasmanian pademelons (*Thylogale billardierii*).

Model	Life stage	Coefficients						r^2^	RMSE
**Linear**		**Intercept**	**a**					0.975	4.68
	P	0.864±0.041	0.02452					0.975	4.32
**Logistic**		**A**	**K**	**L_0_**			r		
	P	−8.88±1 6.36	118.68±32	22.98±14.7			0.33±0.1	0.98	2.30
**Brody**		**B**	**C**		**k**				
Male	PP	133.83±2.35	0.679±0.09		0.068±0.019			0.66	7.75
Female	PP	130.61±2.80	0.44±0.01		0.029±0.007			0.44	9.19
**Brody**		**a**	**b**		**k**				
Male	E	9.8±9.32	127.0±8.16		6.01e-01±0.04			0.48	15.17
Female	E	9.0±4.24	165.0±5.32		7.92.e-02±0.07			0.58	14.2
**SJ2P**		**C**	**K**	**L_0_**	**k**	**j**	**r**		
Male	E	0.44±0.012	92.12±3.7	13.38±0.09	0.065±0.001	8.31±0.04	0.45±0.02	0.90	11.16
Female	E	0.42±0.3	88.92±9.2	11.76±0.91	0.026±0.002	11.64±0.05	0.47±0.01	0.88	9.64

Coefficients of the Brody model describe head length growth after pouch vacation of Tasmanian pademelons (*Thylogale billardierii*).

Parameter B is the mature (or asymptotic) head length, C is an adjustment parameter when *L0*≠*0* or *t*≠*0*, and k is the maturing rate index representing the ratio of maximum growth rate to mature size. For life stage, P represents the model that describes the pouch life stage and PP represents the model that describes the post-pouch life stage. Goodness of fit is given by r^2^: r-square value of the regression, and RMSE is the root mean square error (see Table 1).

## Discussion

This research demonstrates a new methodology for producing a single smooth-joining growth model for describing the sex-specific lifetime growth of a macropod, the Tasmanian pademelon *Thylogale billardierii*. The lifetime growth of pademelons could not be accurately described using previously established single function growth models [10]. Instead, the lifetime growth of both sexes of the pademelon were better described by a curve obtained by a smooth joining of the Verhulst logistic function, which describes the pouch phase growth, and the Brody function, which describes the post-pouch growth phase. We have called this model the smooth-joining two-phase growth model (SJ2P). The novelty of this methodology is that it joins two separate models smoothly. The join point is chosen to minimize the residual sum of squares, rather than choosing the join point based on visual judgment.

The main aim of describing animal growth over time is to provide a practical means of ageing wild animals for life history studies and for quantifying age-specific vital rates that provide the basis for robust wildlife management [15]. To provide accurate estimates of age in macropods, previous methods have used a four parameter broken-stick model [10]. However, some have cautioned against the use of such models because they may not be biologically realistic [10]. Our novel approach improves on those described previously because our model describes growth as continuous through the join point of the two (pre- and post-weaning) functions, so that life-time growth is described as single continuous curve.

The two problems in fitting growth curves to data such as these are (1) the abrupt life-history transition between pouch and post-pouch phases and (2) variability in the morphometric data. For animals with disparate growth phases over their lifetime, including macropods and generally most eutherians, a single function describing lifetime growth is unlikely to represent growth accurately [6]. In macropods, there is an abrupt change in growth rate once the animal leaves the pouch (i.e. weaning), so that post-weaning growth is often better depicted by a different function to that describing pre-weaning growth [11]. The relatively abrupt growth rate change reflects the ecological and behavioural shifts of the animals associated with the adult or post-pouch phase of the life-history. Once animals exit the pouch environment they must allocate more resources to support independent foraging and predator avoidance, whilst being subjected to the limits placed on them by fluctuating environmental conditions [7], [8]. Hence, body mass and morphometric characteristics will vary across a population and within cohorts which creates difficulties when trying to fit growth functions that describe the lifetime growth of a species. The variability in morphometrics of post-pouch pademelons was also responsible for the poor fit of the single functions describing lifetime growth. Indeed, the high morphometric variability recorded in animals of a same estimated age may be largely responsible for the early and unrealistic extinction of the fitted growth curves.

Many macropods are seen as pest animals in agricultural-forest landscape mosaics [21] and as such vast amounts of resources are spent on managing populations, often with little information on age-specific vital rates and performance. The nonlinear function that we have developed provides a novel and first practical advance, which provides a tool to estimate animal age for practices including management exercises in the study of wild macropods (e.g. culling regimes), which provide access to cross-sectional data which is invaluable in the management of wildlife [15].

Cross-sectional population data are a summary of the structure of an animal population and consequently, they can exhibit marked variability in life-history attributes (e.g. lifetime reproductive success, morphometric variability, survival probability) that are driven by a combination of intrinsic and extrinsic factors acting on the population over time [33]. For example fluctuations in resources, driven by environmental variability, can influence reproductive rates thus produce intercohort variability in abundance and perhaps differential vital rates. Given this variability, morphometric measurements would be expected to be highly variable because they encapsulate past environmental variability that may affect growth. In itself, this is not surprising, but it is important because the variability in morphometric measurements is a complicating factor in fitting curves to the observed data, so that defining lifetime growth rates becomes difficult. This difficulty may be addressed by assessing the longitudinal growth of individual animals which can result in high precision curve fitting often by a single nonlinear function [32]. However, fitting individual longitudinal growth curves to wild animals is difficult and research is often constrained by using summary (i.e. cross-sectional) data. The authors acknowledge that combining pre-pouch data obtained two decades prior to the collection of post-pouch data [26] requires cautious biological interpretation of model predictions if growth has changed over time, but the purpose of this research was to illustrate methodology for ageing wild animals with disparate growth phases and provide a platform for additional model development in this field. These data are nonetheless biologically informative because they illustrate clearly, by the variability of the data, that environmental conditions are affecting growth rates and hence the condition that animals are in, which in turn dictates individual survival probabilities [34]. Survival is a key life-history trait and variations in survival, especially juvenile survival, can profoundly affect population growth [34], [35] and thus influence wildlife management strategies. Quantifying the environmental drivers of growth and survival are therefore important tools for wildlife management because they determine the rates at which populations grow and utilize their habitats [14].

Here we have presented a novel and relatively simple method that can be used to fit growth curves to the types of (cross-sectional) data most frequently available to wildlife researchers and ecologists. However, to be used effectively as a tool for ageing animals, the second growth phase of this model needs to be re-parameterised with additional longitudinal morphometric data from individual animals. The longitudinal data would improve model fit and thus the accuracy of growth characterisation and age estimates. It is expected that additional data would likely produce a greater central tendency weight that could not be clearly detected in this study, where cross sectional data were scarce for older individuals. As older individuals are likely to have experienced various environmental and intrinsic biological conditions throughout their life time (including reproduction, pregnancy, intraspecific competition, reduced or abundant sources of food), these factors are likely to affect their growth and thus their biometric measurements. Older adults animal are thus more likely to exhibit considerable variability in their body measurements, compared to younger individuals, which makes it difficult to age them using biometric modelling.

The smooth-joining methodology presented here provides an advance in studying and managing wildlife populations because it allows researchers and wildlife managers to fit a descriptive curve to variable morphometric data that, in general, fits and exemplifies the lifetime growth of animals more accurately than a single nonlinear function. With additional development of the second growth phase of this model, this approach will allow the more precise detection, evaluation and quantification of key changes in body growth which are often associated with the key life-history stages and population growth parameters such as the onset of reproduction [3], [36], and we offer a more suitable platform from which such improvements can be made through further model development. We have used a Verhulst logistic function for the first growth phase, followed by the Brody equation for the second growth phase. In principle, any two suitable curves might be used for the respective phases. The methodology may be compared with the bent cable models that were described previously [37]. These use a quadratic curve to join two lines, where we have joined two non-linear functions without the use of anything akin to the quadratic.

## Supporting Information

Appendix S1 R code for pademelon growth models and data plotting (also available from the URL http://www.maths.anu.edu.au/~johnm/pubs/pademelon).
(DOCX)
