# Supplementary material for: A Two-Phase Model for Smoothly Joining Disparate Growth Phases in the Macropodid Thylogale billardierii
Source: PLoS One. 2011 Oct 12;6(10):e24934. doi: 10.1371/journal.pone.0024934 (PMC3192044; doi:10.1371/journal.pone.0024934)
Supplement: Appendix S1 — R code for pademelon growth models and data plotting (also available from the URL http://www.maths.anu.edu.au/~johnm/pubs/pademelon). (DOCX) [file pone.0024934.s001.docx]

**Appendix S1**

R Code is available from the URL <http://www.maths.anu.edu.au/~johnm/pubs/pademelon>

**#Open datafiles /create subsets of data**

**#Pes length data**

**#Males**

setwd(dirname(file.choose()))

rosem<-read.csv("rosem.csv",sep=";",na.string="NA",dec=",")

d<-subset(rosem, AGE<10)

**#Females**

setwd(dirname(file.choose()))

rosef<-read.csv("rosef.csv",sep=";",na.string="NA",dec=",")

e<-subset(rosef, AGE<10)

**#Head length data**

**#Males**

setwd(dirname(file.choose()))

headm<-read.csv("headm.csv",sep=",",na.string="NA",dec=",")

headm$HL<-as.numeric(as.vector(headm$HL))

headm$age<-as.numeric(as.vector(headm$age))

m<-subset(headm, age<10)

**#Females**

setwd(dirname(file.choose()))

headf<-read.csv("headf.csv",sep=",",na.string="NA",dec=",")

headf$HL<-as.numeric(as.vector(headf$HL))

headf$age<-as.numeric(as.vector(headf$age))

f<-subset(headf, age<10)

**# Model first phase of growth for both sexes (Logistic growth/pouch life)**

**# Verhulst logistic growth : Pes length (first growth phase) age <10**

logist <- LL ~ A + K * L0/(L0+ (K - L0) * exp(-r * AGE))

logist.nls <- nls(logist, subset(rosem, AGE<8),

start=list(A=15, K=100, L0=1.5, r=.9))

**# Verhulst logistic growth : head length (first growth phase) age <10**

logisth <- HL ~ A + K * L0/(L0+ (K - L0) * exp(-r * age))

logisth.nls <- nls(logisth, subset(headm, age<8),

start=list(A=15, K=100, L0=1.5, r=.9))

**# Figure 2  a & b : Logistic growth Pes & Head length (pouch life)**

**#double plot**

tiff(file = "temp.tiff", width = 6400, height = 3200, units = "px", res = 800)

close.screen(all=true)

split.screen(c(1,2))

**screen(1)**

plot(LL ~ AGE, data=rosem, subset=AGE<8, xlab="Age (months) ",ylab="Pes length(mm)", ylim=c(20,100), cex=0.5)

hatc <- fitted(logist.nls)

with(subset(rosem, AGE<8),

points(AGE, hatc, col=4, pch=12, cex=0.5))

legend(0.5,100,"A",bty="n")

**screen(2)**

plot(HL ~ age, data=m, subset=age<8, xlab="Age (months) ",ylab="Head length length(mm)", ylim=c(20,100), cex=0.5)

hath <- fitted(logisth.nls)

with(subset(headm, age<8),

points(age, hath, col=4, pch=12, cex=0.5))

legend(0.5,100,"B",bty="n")

dev.off()

**# Model second phase of growth for males & females (post pouch-life/ Brody)**

**# Brody growth second growth phase PES LENGTH MALES**

brody <- LL ~ B*(1 -C*(exp(-k*AGE))) # brody

rose2m.brody <- nls(brody, subset(rosem, AGE>=8), start=list(B=150, C=0.5, k=0.1))

rose2m.brody

**# Brody growth second growth phase PES LENGTH FEMALE**

brody <- LL ~ B*(1 -C*(exp(-k*AGE))) # brody

rose2f.brody <- nls(brody, subset(rosef, AGE>=8), start=list(B=150, C=0.5, k=0.1))

rose2f.brody

**# Fig 2  c & d : Sex-dependant second growth phase (Brody function)**

**#double plot**

close.screen(all=TRUE)

tiff(file = "temp.tiff", width = 6400, height = 3200, units = "px", res = 800)

split.screen(c(1,2))

**screen(1)**

plot(LL ~ AGE, data=rosem, subset=AGE>=8,xlab="Age (months) ",ylab="Pes length (mm)", pch=20, cex=0.4, ylim=c(100,190))

hatb <- fitted(rose2m.brody)

with(subset(rosem, AGE>=8),

points(AGE, hatb, col="red", cex=0.6,pch=10))

legend(5,190,"C",bty="n")

**screen(2)**

plot(LL ~ AGE, data=rosef, subset=AGE>=8,xlab="Age (months) ", pch=20, cex=0.4,ylim=c(100,190), ylab="")

hate <- fitted(rose2f.brody)

with(subset(rosef, AGE>=8),

points(AGE, hate, col="red", cex=0.6, pch=10))

legend(5,190,"D",bty="n")

dev.off()

**# Brody growth fitted over entire life PES LENGTH MALES**

brody <- LL ~ B*(1 -C*(exp(-k*AGE))) # brody

rose2m.brody <- nls(brody, rosem, start=list(B=150, C=0.5, k=0.1))

rose2m.brody

**# Brody growth fitted over entire life PES LENGTH FEMALE**

brody <- LL ~ B*(1 -C*(exp(-k*AGE))) # brody

rose2f.brody <- nls(brody, rosef, start=list(B=150, C=0.5, k=0.1))

rose2f.brody

**# Figure 3 : BRODY GROWTH ENTIRE LIFE PES LENGH**

**#double plot**

close.screen(all=true)

tiff(file = "temp.tiff", width = 6400, height = 3200, units = "px", res = 800)

split.screen(c(1,2))

**screen(1)**

hatb <- fitted(rose2m.brody)

adm <- data.frame(AGE= pretty(rosem$AGE,40))

adm$hatb<- predict(rose2m.brody, newdata=adm)

plot(LL ~ AGE, data=rosem ,xlab="Age (months) ",ylab="Pes length (mm)", pch=21, ylim=c(0,190), cex=0.5)

lines(hatb ~ AGE,data=adm, col= "red", lwd=2, lty=2)

legend(0,190,"A",bty="n")

**screen(2)**

hate <- fitted(rose2f.brody)

ade <- data.frame(AGE= pretty(rosef$AGE,40))

ade$hate<- predict(rose2f.brody, newdata=ade)

plot(LL ~ AGE, data=rosef ,xlab="Age (months) ",ylab="", pch=21, ylim=c(0,190), cex=0.5)

lines(hate ~ AGE,data=ade, col="red", lwd=2, lty=2)

legend(0,190,"B",bty="n")

dev.off()

**# SJ2P Model : Both non-linear functions forced through a defined age parameter & Fig 4**

hatboth <- function(AGE, A, K, L0, r, B, C, k, phase){(1-phase)*(A + K * L0 * exp(r * AGE)/(K + L0 * (exp(r * AGE) - 1))) + phase*(B*(1 -C*(exp(-k*AGE)))) }

logist <- LL ~ A + K * L0/(L0+ (K - L0) * exp(-r * AGE)) function used before

**# Combine logistic and Brody**

both <- function(AGE, K, L0, r, B, C, k){

ifelse(AGE<10, K * L0/(L0+ (K - L0) * exp(-r * AGE)) - K * L0/(L0+ (K - L0) * exp(-r * 10)) +

B*(1 -C*(exp(-k*10))), B*(1 -C*(exp(-k*AGE))))

}

**# Males**

male.nls <- nls(LL ~ both(AGE, K, L0, r, B, C, k),

start=list(K=100, L0=2, r=.6, B=155, C=.6, k=.078), data=rosem)

adm <- data.frame(AGE= pretty(rosem$AGE,40))

adm$hat <- predict(male.nls, newdata=adm)

***# arrow plot***

close.screen(all=true)

tiff(file = "temp.tiff", width = 10400, height = 3200, units = "px", res = 800)

split.screen(c(1,3))

**screen(1)**

plot(LL ~ AGE, data=rosem, subset=AGE<8, xlab="Age (months) ",ylab="Pes length(mm)",ylim=c(0,200),xlim=c(0,80), cex=0.5)

hath<- fitted(logist.nls)

with(subset(rosem, AGE<8),

points(AGE, hath, col=4,cex=0.4, pch=12))

legend(0,190,"a1",bty="n")

**screen(2)**

plot(LL ~ AGE, data=rosem, subset=AGE>=8,xlab="Age (months) ",ylab="", ylim=c(0,200),xlim=c(0,80), cex=0.5)

hatb <- fitted(rose2m.brody)

with(subset(rosem, AGE>=8),

points(AGE, hatb, col=5, pch=12, cex=0.4))

legend(0,190,"a2",bty="n")

**screen(3)**

plot(LL ~ AGE, data=rosem, ylab="",xlab="Age (months)", cex=0.5)

lines(hat ~ AGE, data=adm, lwd=2, lty=2, col="red")

legend(0,190,"a3",bty="n")

dev.off()

**# Females**

female.nls <- nls(LL ~ both(AGE, K, L0, r, B, C, k),

start=list(K=100, L0=2, r=.6, B=155, C=.6, k=.078), data=rosef)

ade<- data.frame(AGE= pretty(rosef$AGE,40))

ade$hat <- predict(female.nls, newdata=ade)

***## arrow plot***

close.screen(all=true)

tiff(file = "temp.tiff", width = 10400, height = 3200, units = "px", res = 800)

split.screen(c(1,3))

**screen(1)**

plot(LL ~ AGE, data=rosem, subset=AGE<8, xlab="Age (months) ",ylab="Pes length(mm)",ylim=c(0,200),xlim=c(0,80), cex=0.5)

hath<- fitted(logist.nls)

with(subset(rosem, AGE<8),

points(AGE, hath, col=4,cex=0.4, pch=12))

legend(0,190,"b1",bty="n")

**screen(2)**

plot(LL ~ AGE, data=rosef, subset=AGE>=8,xlab="Age (months) ",ylab="", ylim=c(0,200),xlim=c(0,80), cex=0.5)

hate<- fitted(rose2f.brody)

with(subset(rosef, AGE>=8),

points(AGE, hate, col=5, pch=12, cex=0.4))

legend(0,190,"b2",bty="n")

**screen(3)**

plot(LL ~ AGE, data=rosef, ylab="",xlab="Age (months)", cex=0.5,ylim=c(0,200),xlim=c(0,80))

lines(hat ~ AGE, data=ade, lwd=2, lty=2, col="red")

legend(0,190,"b3",bty="n")

dev.off()

**# Make derivatives at AGE=critage agree; estimate critage; critage= actual age where the growth changes & fig 5**

tiff(file = "temp.tiff", width = 3200, height = 3200, units = "px", res = 800)

equalb <- function(AGE, K, L0, r, C, k, critage){

B <- exp(k*critage)*r * K * L0 *(K-L0)*exp(-r*critage)/(L0 + (K-L0)*exp(-r * critage))^2/(k*C)

ifelse(AGE<critage, K * L0/(L0+ (K - L0) * exp(-r * AGE)) -

K * L0/(L0+ (K - L0) * exp(-r * critage)) +

B*(1 -C*(exp(-k*critage))),

B*(1 -C*(exp(-k*AGE))))

}

eqmb.nls <- nls(LL ~ equalb(AGE, K, L0, r, C, k, critage),

start=list(K=98.2, L0=1.68, r=.882, C=.657, k=.0857, critage=10), data=rosem)

tiff(file = "temp.tiff", width = 3200, height = 3200, units = "px", res = 800)

plot(LL ~ AGE, data=rosem,cex=0.5,col="blue", ylab="Pes length",xlab="Age (months)")

ad <- data.frame(AGE= pretty(rosem$AGE,40))

ad$mhat <- predict(eqmb.nls, newdata=ad)

lines(mhat ~ AGE, data=ad, col="blue", lwd=2, lty=2)

**# Now add females**

eqfb.nls <- nls(LL ~ equalb(AGE, K, L0, r, C, k, critage),

start=list(K=108, L0=3.45, r=.74, C=.24, k=.03, critage=10),

data=rosef)

with(subset(rosef, AGE>=10), points(LL~ AGE, col="red",cex=0.5))

ad$fhat <- predict(eqfb.nls, newdata=ad)

lines(fhat ~ AGE, data=ad, col=2, lwd=2, lty=2)

dev.off()
